# Consolidation and Forced Elasticity in Double-Network Hydrogels

**DOI:** 10.3390/gels9030258

**Published:** 2023-03-22

**Authors:** S. Shams Es-haghi, R. A. Weiss

**Affiliations:** 1 Advanced Structures and Composites Center, The University of Maine, Orono, ME 04469-5793, USA; 2 Department of Polymer Engineering, The University of Akron, 250 S. Forge St., Akron, OH 44325-0301, USA

**Keywords:** double-network hydrogels, finite tensile deformation, confined compression, forced elasticity, consolidation, diffusion

## Abstract

This paper discusses two observations that are unique with respect to the mechanics of double network (DN) hydrogels, forced elasticity driven by water diffusion and consolidation, which are analogous to the so-called Gough–Joule effects in rubbers. A series of DN hydrogels were synthesized from 2-acrylamido-2-methylpropane sulfuric acid (AMPS), 3-sulfopropyl acrylate potassium salt (SAPS) and acrylamide (AAm). Drying of AMPS/AAm DN hydrogels was monitored by extending the gel specimens to different stretch ratios and holding them until all the water evaporated. At high extension ratios, the gels underwent plastic deformation. Water diffusion measurements performed on AMPS/AAm DN hydrogels that were dried at different stretch ratios indicated that the diffusion mechanism deviated from Fickian behavior at extension ratios greater than two. Study of the mechanical behavior of AMPS/AAm and SAPS/AAm DN hydrogels during tensile and confined compression tests showed that despite their large water content, DN hydrogels can retain water during large-strain tensile or compression deformations.

## 1. Introduction

Hydrogels are crosslinked polymers that can be highly swollen with water (many hundred percent). Examples of their use include the water-absorbing ingredient of diapers, cosmetics, food (e.g., Jello), sensors and biomaterials, such as scaffolds for tissue engineering and artificial organs [1,2,3,4]. Most hydrogels are brittle, largely because of high internal stresses in the polymer network due to high swelling of the polymer by water. About 20 years ago, Gong et al. [5] discovered that extraordinarily tough hydrogels could be made by sequential free-radical polymerization of two hydrophilic polymer networks, which they termed double-network (DN) hydrogels. The microstructure of a DN hydrogel was originally believed to be an interpenetrating network (IPN). Tough DN hydrogels can also be achieved with the sequential polymerization approach without using a crosslinking monomer in the second polymerization, and the microstructure of those materials is referred to as a semi-interpenetrating network, SIPN. Infrared spectra analyses, however, indicated that the synthetic approach used by Gong et al. [5] also produced some covalent grafting between the first and second networks, and that was necessary for achieving extraordinary toughness [6]. Therefore, in this paper, the DN hydrogels produced with or without crosslinking in the second polymerization step are referred to as pseudo-IPN or pseudo-SIPN, respectively. 

When a hydrogel is deformed, water is released. [7]. Conventional single-network hydrogels usually have poor mechanical properties and premature failure of these materials at low strains prevents studying the diffusion of water out of the gel at large deformations. DN hydrogels, however, can undergo large-strain elasto-plastic deformations, even with large water concentrations (~90% of the gel) [8]. High water content and high extensibility make DN hydrogels an interesting material for studying the diffusion of water from a gel during finite deformations. For tensile deformations with very low extension rates, a significant amount of water may evaporate from the sample during the experiment. If enough water evaporates, the sample may actually vitrify and freeze-in extended conformations of the network chains. Vitrification may also occur at high extension rates when the sample is held in a highly extended state for a time long enough for the water to evaporate [9].

In this paper, drying of DN hydrogels under an external tensile load was evaluated. The objective of the study was to understand the kinetics of diffusion of water and re-equilibration of the dried extended DN hydrogel in water, as well as the effect of external force on expelling water from the gels under large deformations. Two interesting observations were made regarding the mechanics of DN hydrogels that are analogous to Gough–Joule effects in rubbers. 

## 2. Results and Discussion

### 2.1. Forced Elasticity Driven by Diffusion of Water 

**Figure 1:**
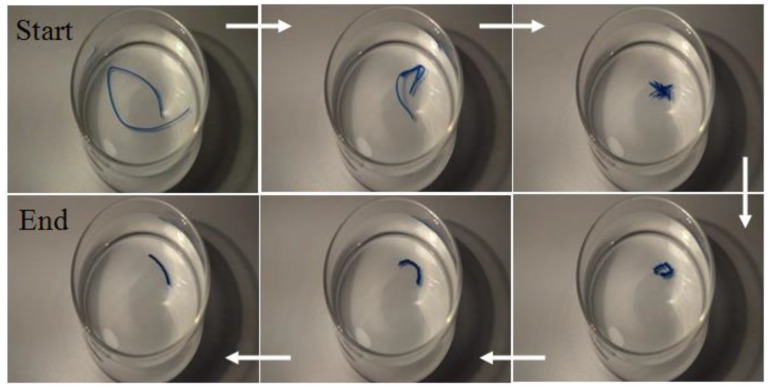
The forced elasticity driven by diffusion of water into a DN hydrogel dried in its extended state. “Start” denotes when the dry extended hydrogel is placed back into water. The arrows show the temporal progression of the shape of the hydrogel, and “End” denotes the point where a new regime of diffusion starts towards a new equilibrium state. Times associated with each image in the direction of arrows are *t* = 0, 7, 13, 45, 51 and 56 s.

When a DN hydrogel is extended by the application of a tensile stress and the deformation is maintained until all the water in the sample evaporates, the resulting dry material is a glass, but one where the chains are frozen in an extended state. When the dry extended hydrogel is swollen with water again, the sample quickly contracts and gradually reaches a point where the sample stops moving (Figure 1), and a new regime of diffusion towards a new equilibrium swollen state starts. As will be shown later in this paper, the time before retraction begins and the onset of stress release are strongly dependent on the stretch ratio at which the sample was dried.

The observation reported herein is similar to what is observed in polymers undergoing large deformations via cold-drawing. In effect, the high deformation of cold-drawn polymers is recoverable in glassy polymers upon heating the sample to a sufficiently high temperature below the decomposition temperature or the melting temperature for semi-crystalline polymers [10]. This thermo-recoverability is a form of forced elasticity driven by heat flux. The retraction of the dried extended DN hydrogels after immersion in water is another form of forced elasticity which is driven by solvent flux, in this case, water. 

When all the water evaporates from a stretched DN hydrogel, the glass transition of the sample becomes very high, which reduces the mobility of the polymer chains that have been frozen in extended, entropically unfavorable conformations. When the sample is rehydrated, the polymer chains return to their more entropically favorable, original random-coil conformations. As will be shown later in this paper, the higher the extension of the polymer chains, the quicker the retraction process commences. In effect, the forced elasticity in DN hydrogels is a relaxation process in which the stresses frozen in the sample relax upon diffusion of water into the material. In this relaxation process, water acts as a plasticizer that significantly reduces the glass transition of the dried gel. The forced elasticity driven by water diffusion can be compared to the first Gough–Joule effect in thermoelasticity of rubbers, in that a rubber stretched under a constant load, contracts reversibly upon heating [11]. 

Upon immersing the dried, stretched DN hydrogel in water, two regimes of diffusion are apparent. The first regime is a fast uptake of water driven by the residual stress frozen into the material that relaxes to zero (stress-assisted diffusion) (see Figure 1), and the second regime is a slow diffusion process driven by osmotic stress (i.e., a chemical potential gradient due to differences in the water concentrations inside and outside the gel). The onset of the second regime of diffusion may be considered as the time at which the sample stops moving in water. The rapid diffusion process and stress relaxation in the first regime of diffusion makes accurate measurement of water content very difficult during this regime. Consequently, diffusion measurements were performed only in the second regime of diffusion. 

The samples for the diffusion experiments were DN hydrogels previously hydrated with water to equilibrium. These samples were then stretched to different stretch ratios and held at constant strain while the water completely evaporated. During evaporation, the stress in the sample was monitored. That procedure provided dry DN samples with frozen-in stress extended at different stretch ratios. The dried samples were then returned to a container of water, and the second regime of the diffusion process was measured gravimetrically (i.e., by periodically weighing the sample). The kinetics of the water sorption was described by Equation (1) [12]:

(1) $$\frac{M_{t}}{M_{\infty }}=kt^{n}$$

where $M_{t}$ and $M_{\infty }$ represent the mass uptakes at time *t* and at equilibrium, *k* is a constant and *n* characterizes the mechanism of diffusion. 

**Figure 2:**
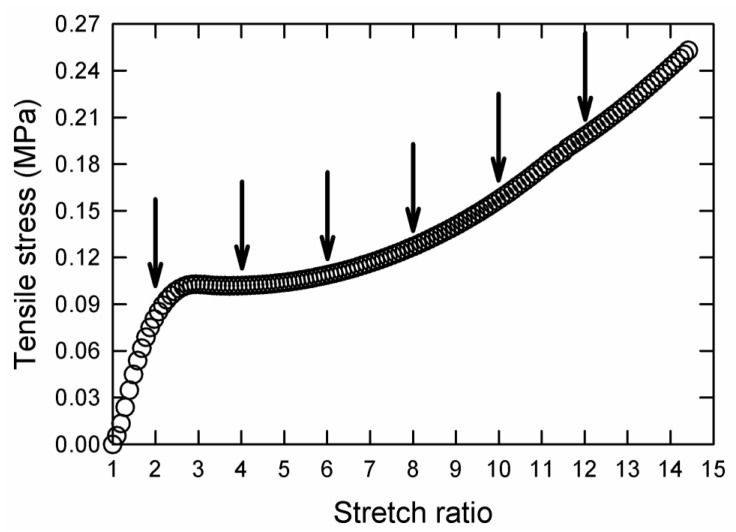
Engineering tensile stress versus stretch ratio for a pseudo-IPN hydrogel made of AMPS(1,2,2,9) and AAm(2,0.1,0.01,97). Arrows display the stretch ratios at which drying tests were performed on the samples.

Figure 2 shows the tensile deformation behavior of a pseudo-IPN AMPS(1,2,2,9)/AAm(2,0.1,0.01,97) DN hydrogel (see “Experimental Section” for a description of the materials used in this investigation). Drying tests were performed by stretching the DN hydrogel with an extension rate of 50 mm/min to different stretch ratios shown by arrows in Figure 2 and holding it at those stretch ratios until all the water evaporated. 

**Figure 3:**
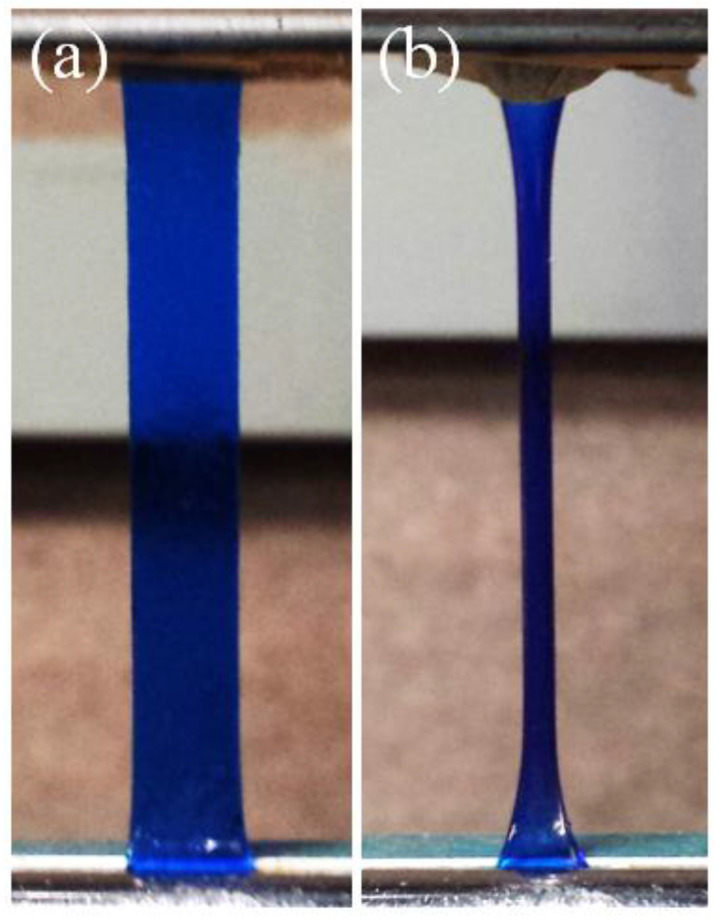
AMPS(1,2,2,9)/AAm(2,0.1,0.01,97) DN hydrogel. (**a**): after being extended to λ = 2; (**b**): after being dried overnight in the extended form.

Figure 3 shows the AMPS(1,2,2,9)/AAm(2,0.1,0.01,97) DN hydrogel sample after being extended to λ=2 ( Figure 3a) and after all the water evaporated under extension (Figure 3b). Methylene blue was used to dye the sample to make it more noticeable. It should be noted that for drying experiments, no methylene blue dye was used.

**Figure 4:**
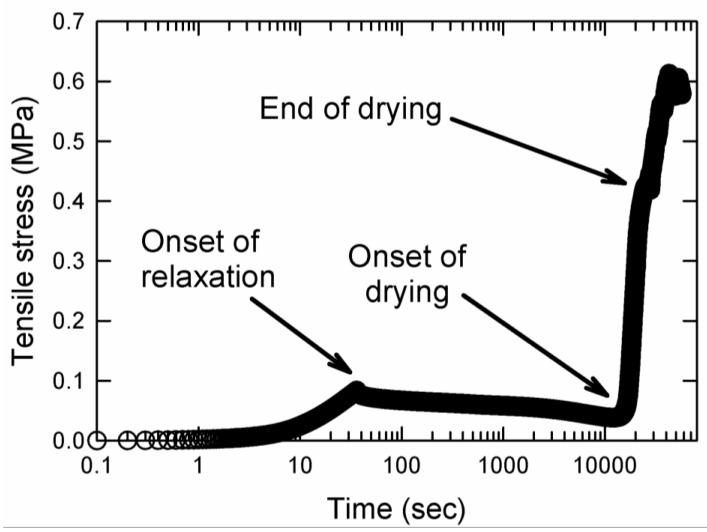
Drying experiment for AMPS(1,2,2,9)/AAm(2,0.1,0.01,97) DN hydrogel stretched to λ = 2. The arrows denote the onset of relaxation, onset of drying and end of drying.

Figure 4 shows the result of the drying experiment for a AMPS(1,2,2,9)/AAm(2,0.1,0.01,97) sample extended to λ=2. The onset of relaxation, onset of drying and end of drying are marked on the graph in Figure 4. During stress relaxation, any changes in the stress were negligible. Note that the onset of drying was characterized by a rapid increase in the stress.

**Figure 5:**
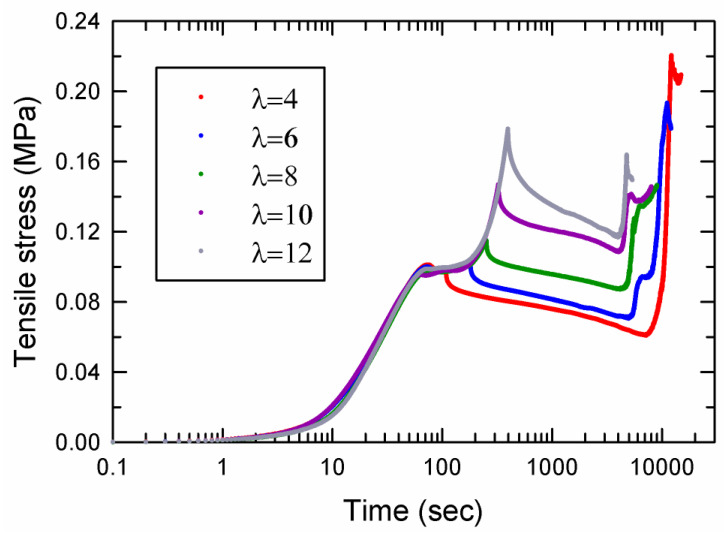
Results of drying experiments (tensile stress versus time) for AMPS(1,2,2,9)/AAm(2,0.1,0.01,97) DN hydrogels with various stretch ratios.

Figure 5 shows the results of drying experiments for AMPS(1,2,2,9)/AAm(2,0.1,0.01,97) DN hydrogels with various extension ratios. All of the samples exhibited the same general trend that was observed for the drying of the sample with λ=2 in Figure 4. For the sample stretched to λ=6, a small kink was observed during the drying period where the stress rapidly increased, shown by the blue curve in Figure 5. The kink is due to the non-uniform thickness of the sample at that stretch ratio. A substantial part of that sample became thin due to propagation of a neck, which resulted in quicker drying of that part of the sample. In general, by increasing the stretch ratio at the onset of relaxation, the onset of drying shifted to earlier times.

**Figure 6:**
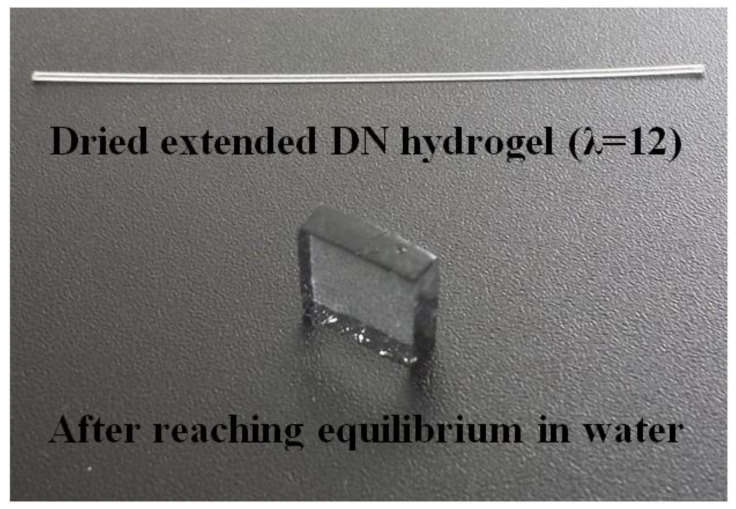
AMPS(1,2,2,9)/AAm(2,0.1,0.01,97) DN hydrogel; top: a segment of dried gel stretched up to λ = 12; bottom: the top dried gel rehydrated to the equilibrium swelling ratio.

Water sorption experiments were conducted with the dried samples of the AMPS(1,2,2,9)/AAm(2,0.1,0.01,97) DN hydrogels stretched to the different extension ratios. Figure 6 shows a segment of the dried hydrogel sample stretched to λ=12 and after being rehydrated to the equilibrium swelling ratio.

**Figure 7:**
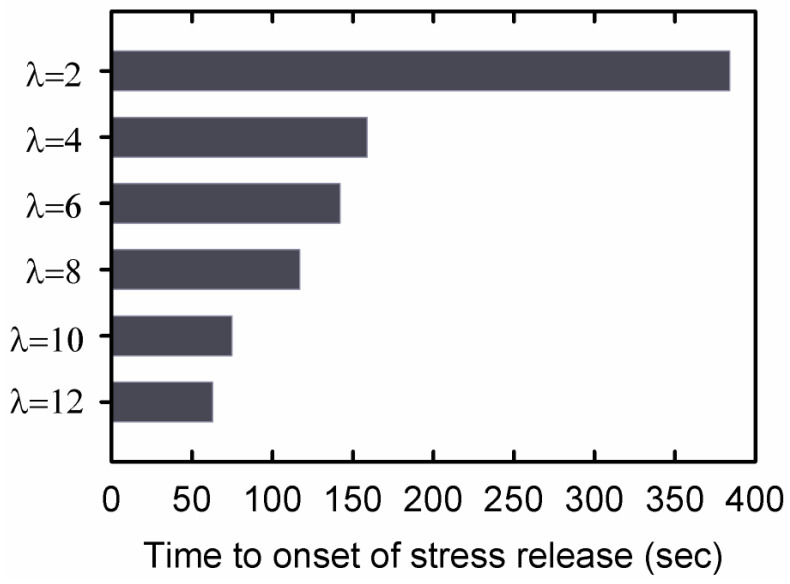
The time to onset of stress release (TOSR) for the dried AMPS(1,2,2,9)/AAm(2,0.1,0.01,97) DN hydrogels with different extension ratios. TOSR was defined as the time it took for the stretched sample to begin bending when immersed in water.

The time to onset of stress release (TOSR) was defined as the time it took for a stretched, dried DN hydrogel to begin bending when the hydrogel was immersed in water. Figure 7 compares the TOSR for the AMPS(1,2,2,9)/AAm(2,0.1,0.01,97) DN hydrogels with different stretch ratios. The TOSR decreased with increasing stretch ratio. 

The sample extended to λ=2, which is associated with the extension of the sample before plastic deformation (see Figure 2), showed the highest TOSR. By increasing the stretch ratio to λ=4, where the gel sample experienced plastic deformation, a large reduction in TOSR was observed. For higher extension ratios, plastic deformation occurred, and the TOSR decreased. 

**Figure 8:**
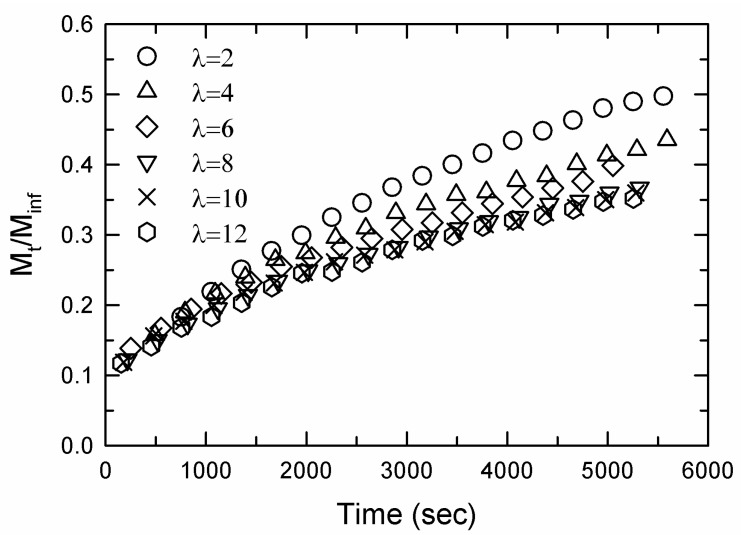
Water diffusion data for the AMPS(1,2,2,9)/AAm(2,0.1,0.01,97) DN hydrogel. The sample with λ = 2 exhibited Fickian diffusion, but for λ > 2 the diffusion became increasingly non-Fickian.

**Figure 9:**
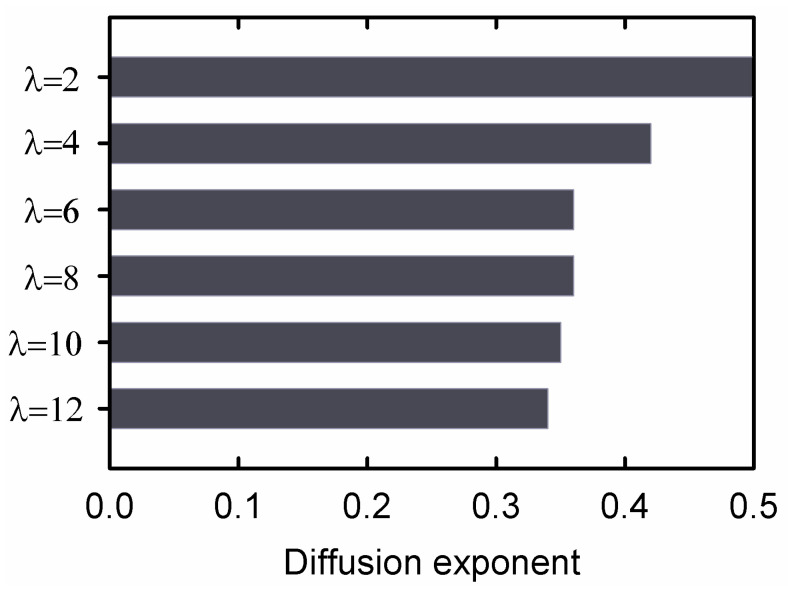
Exponents derived from diffusion measurements. A value of *n* = 0.5 indicates Fickian diffusion.

Figure 8 shows the water diffusion results for the second regime of diffusion. The diffusion exponent, *n*, was determined from a linear least square fit of Equation (1) to the data, and the results are shown in Figure 9. For the sample with λ=2, *n* = 0.5, which indicated Fickian diffusion. Deviation from Fickian diffusion increased with increasing extension ratio.

The non-Fickian diffusion behavior shown in Figure 8 for hydrogel samples stretched to λ > 2 is a consequence of diffusion in a relaxing medium [13]. The very fast contraction of the sample in the first regime of diffusion for samples stretched to high extension ratios indicates that the sample is in a non-equilibrium state. Extension of a DN hydrogel to high stretch ratios results in damage to the internal structure of the material, and the severity of the damage increases when increasing the stretch ratio [8]. As a result, the crosslink density is reduced in samples extended to a high stretch ratio compared to that in the original sample. In the first regime of diffusion, frozen stresses are released, but the diffusion process is too fast to allow the polymer chain strands to relax completely to their equilibrium conformations. The non-Fickian water diffusion is a consequence of the relaxation process that occurs simultaneously with the water absorption process. The swelling process produces expansion of the gel, which facilities relaxation of the gel to a new equilibrium state. Increasing the stretch ratio of the dry hydrogel decreases the crosslink density, increases the amount of water the stretched gel can absorb and perturbs the diffusion process further from Fickian behavior. The variation of TOSR shown in Figure 7, which measures the onset of fast contraction in the first regime of diffusion, is consistent with the decreasing value of the diffusion exponent in Figure 9. 

### 2.2. Consolidation

**Figure 10:**
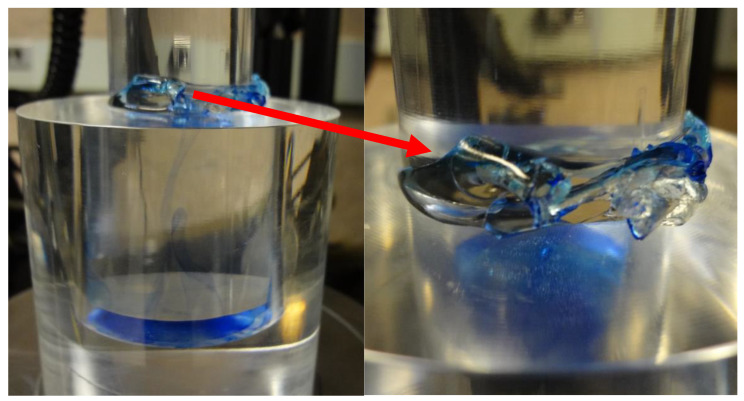
Confined compression of SAPS(1,2,2,9)/AAm(2,0.1,0,97) DN hydrogel. The hydrogel retains water under confined compression, but when the compression becomes too high, some parts from the edges of the sample climb up the gap. This behavior was observed in all tough DN hydrogels studied.

Consolidation is a term coined by Terzaghi [14] in the field of soil mechanics to describe any process involving a decrease in the water content of saturated soil without replacement of water with air. In solid mechanics (mechanics of porous media), consolidation is a term that describes the diffusion of a liquid out of a solid containing a liquid phase when external stress is applied [15]. Thus, consolidation may have some importance in soft, water-containing materials that can undergo large deformations, such as DN hydrogels. Figure 10 demonstrates the confined compression of a SAPS(1,2,2,9)/AAm(2,0.1,0,97) DN hydrogel (see “Experimental Section” for the gel details). The DN hydrogels retained the water during compression, but at high deformation, some parts from the edges of the sample climbed up the gap between the plunger and the container. In confined compression, the sample is not allowed to deform in the lateral directions upon applying the external load. In effect, if consolidation takes place, the water should diffuse out of the sample.

Mechanical experiments indicate that DN hydrogel can hold water under an external force. 

All the tough DN hydrogels evaluated in these experiments exhibited similar behavior for confined compression. All retained water during compression, but at sufficiently high deformation, all exhibited some material climbing the gap. This behavior contrasts with the behavior of single-network hydrogels, where the application of external stresses produces expulsion of the solvent [7].

The capacity of a chemically crosslinked hydrogel to absorb water depends on the crosslink density of the gel, as well as temperature and pressure. The amount of water is a consequence of a balance between two competing phenomena: (1) an osmotic force that drives sorption and (2) the retractive forces in the network chains that oppose sorption. Equilibrium sorption occurs when the two forces balance. At equilibrium, the gel can absorb no more water. However, a macroscopic deformation of the gel is accompanied by microscopic changes in the conformations of the network chains that increase the retractive forces in the network and push the equilibrium state to a lower water capacity. Because conventional single-network hydrogels have poor extensibility, consolidation is not detected due to premature fracturing of the gel. Consolidation, however, can be studied in the tough DN hydrogels. 

The confined compression results for the DN hydrogels may be related to the unique microstructure of these materials. Water may leave a DN hydrogel via two mechanisms. The first mechanism is the diffusion of water to the surface of the sample, where the water evaporates. This process is driven by the gradient of chemical potential. This is the dominant mechanism for a stress-free DN hydrogel. When the sample is deformed, another contribution might be considered due to the applied external stresses to the material. To study the effect of the microstructure of DN hydrogels on the consolidation, we can consider tough DN hydrogels with physically trapped polymer chains. In a previous study [16], we showed that by using a very low concentration of very high molecular weight PEO chains trapped in the first network, it is possible to synthesize a tough hydrogel, although the second polymerization step was conducted with a very low UV dose. For example, the SAPS(1,4,2,9,PEO)/AAm(2,0.1,0.05,9) DN hydrogel is tough, although the second polymerization step was performed with a very low UV dose.

**Figure 11:**
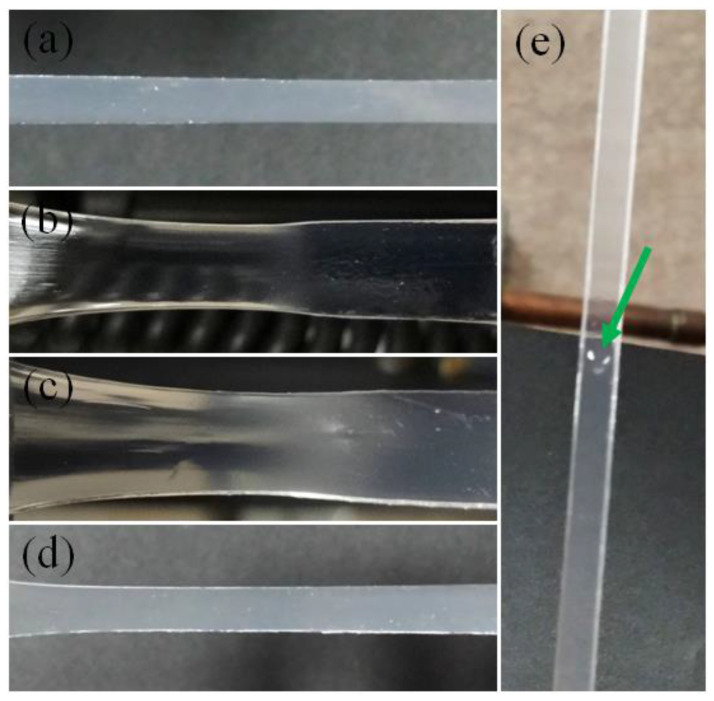
Appearance of DN hydrogels under tensile loading: (**a**): AMPS(1,1,4,9)/AAm(2,0.01,0.01,97); (**b**): SAPS(1,0.1,2,9)/AAm(2,0.1,0,97); (**c**): SAPS(1,2,2,9)/AAm(2,0.1,0,97); (**d**): SAPS(1,2,2,97)/AAm(2,0.1,0.05,97); (**e**): SAPS(1,4,2,9,PEO)/AAm(2,0.1,0.05,9). No consolidation was observed in case of conventional DN hydrogels made of second networks with very high molecular weight chains or chain strands. The sample synthesized by trapped PEO chains shows consolidation. Arrow shows a droplet of water falling down the surface of the gel during tensile loading. The consolidation is negligible, however, as compared to the water content of the gel.

Figure 11 shows the appearance of different DN hydrogels under uniaxial tensile tests performed with the extension rate of 50 mm/min. As one can see, no consolidation occurred in the DN hydrogels synthesized with different recipes. For the SAPS(1,4,2,9,PEO)/AAm(2,0.1,0.05,9) DN hydrogel, a tiny droplet of water appeared on the surface of the sample, although the amount of water expelled was much smaller than the water content of the gel. The consolidation in gels is analogous to the second Gough–Joule effect in that a rubber gives out heat when stretched [11]. In consolidation, water flux plays the role of heat flux in rubbers.

## 3. Conclusions

When all the water is evaporated from a stretched DN hydrogel, the material becomes a glass with the polymer chains frozen in extended, entropically unfavorable conformations. When the sample is returned to water, once the water diffuses into the sample, the polymer chains revert to their original random coil conformations. The contraction of the dried stretched DN hydrogel is driven by diffusion of water, which is termed forced elasticity. After immersing the dried, stretched DN hydrogel in water, two regimes of diffusion occur. The first regime is a fast uptake of water driven by relaxation of the frozen-in residual stresses (stress-assisted diffusion), and the second regime is a slow diffusion process driven by the gradient of chemical potential. 

Drying of DN hydrogels synthesized from 2-acrylamido-2-methylpropane sulfuric acid (AMPS) and acrylamide (AAm) was monitored by extending samples to different stretch ratios and maintaining the extensional deformation until all the water evaporated. Diffusion measurements performed in the second regime of diffusion on DN hydrogels dried at different stretch ratios indicated that the water diffusion mechanism deviated from Fickian behavior when the extension ratio was λ > 2.The time to the onset of stress release (TOSR) at the beginning of the first regime of diffusion decreased significantly as the extension ratio increased. 

Large-strain tensile tests and confined compression tests conducted on DN hydrogels indicated that tough DN hydrogels retain water under finite deformations, i.e., no consolidation occurs during finite deformation of a DN hydrogel. Although there is a natural tendency for consolidation in hydrogels as wet solids, the specific structure of these materials prevents consolidation. In contrast, tough DN hydrogels with low molecular weight polymer chain strands in the second network that contain very low concentration of very high molecular weight physically trapped polymer chains do exhibit consolidation during a tensile test.

Forced elasticity and consolidation in hydrogels are analogous to the Gough–Joule effects in rubber thermoelasticity, where water flux plays the role of heat flux. 

## 4. Experimental Section

### 4.1. Materials

The 2-acrylamido-2-methylpropane sulfuric acid (AMPS), 3-sulfopropyl acrylate potassium salt (SAPS), acrylamide (AAm) and poly(ethylene oxide) and PEO (M_v_~4,000,000 g.mol^−1^) were purchased from Sigma-Aldrich Chemical Co. (Milwaukee, WI, USA) and used as received. The crosslinking monomer N,N′-methylenebis(acrylamide) (MBAA) was purchased from Sigma-Aldrich Chemical Co. (Milwaukee, WI, USA) and was recrystallized from ethanol. A photoinitiator, 2-oxoglutaric acid (OXGA), was obtained from Fluka Chemical Co. and used as received.

### 4.2. Synthesis of Polymeric Networks

DN hydrogels were synthesized via a two-step sequential free-radical polymerization. The first network was prepared by adding MBAA and OXGA to a 1 M solution of AMPS in deionized (DI) water. Dry nitrogen gas was bubbled through the reaction mixture for 5–10 min to remove oxygen, and the solution was injected into a glass mold made of two parallel glass slides, which was then exposed to a 365 nm ultraviolet (UV) light source. The resulting AMPS gel was then immersed into a 2 M solution of AAm in DI water containing the crosslinking monomer and initiator, which had already been deoxygenated with nitrogen gas, until an equilibrium swelling was achieved. The AAm-swollen AMPS gel was then placed between two parallel glass slides and exposed to 365 nm UV light. The resulting DN hydrogel sample was immersed in DI water, which was replaced several times with fresh water to remove any unreacted monomers. Two UV intensities, 3 mW/cm^2^ and 15 mW/cm^2^, were used for the photo-polymerization reactions, and the radiation exposure times for each intensity were 9 h (dose = 97 J/cm^2^) and 10 min (dose = 9 J/cm^2^), respectively. The chemical schemes for the free-radical polymerizations can be found in [6,17].

Pseudo-SIPN hydrogels were synthesized using the same procedure described above without using a crosslinking monomer in the second polymerization step. DN hydrogels were also made from SAPS as the monomer for synthesizing the first network. One pseudo-IPN hydrogel was synthesized from SAPS and AAm by adding 0.01 wt% PEO to the SAPS polymerization reaction mixture.

Each individual network in a hydrogel is described by the name of the monomer used, followed by four numbers in parenthesis that indicate (1) the molar concentration of the monomer in deionized water, (2) the mol% of the photoinitiator with respect to the monomer, (3) the mol% of the crosslinking agent with respect to the monomer and (4) the UV dose used for the reaction (i.e., intensity × time of exposure). Thus, AMPS(1,2,2,9) corresponds to the polymerization of a 1 M AMPS solution using 2 mol % OXGA, 2 mol % MBAA and a UV dose of 9 J/cm^2^. The DN hydrogel with the trapped PEO chain in the first network is shown by SAPS(1,4,2,9,PEO)/AAm(2,0.1,0.05,9). Table 1 shows the details of the recipes used for synthesizing hydrogels. 

### 4.3. Tensile Testing

Uniaxial tensile tests were conducted with an Instron 5567 universal testing machine. A 100 N load cell and a constant crosshead speed of 50 mm/min at 23 °C were used for the experiments. Large-strain tensile deformations were applied on hydrogel samples with rectangular shape (length = 40 mm, width = 10 mm, thickness = 2–3 mm) using a gauge length of 30 mm. To prevent slippage of the specimens in the grips of the tensile machine, sandpaper was used. The tensile results are reported as engineering stress versus stretch ratio, where the engineering stress was obtained by dividing the tensile force by the initial cross-sectional area of the specimen and the stretch ratio, λ = L(t)/L_o_, where L(t) was the instantaneous sample length and L_o_ was the initial sample length. 

### 4.4. Drying Tests

Drying tests were performed on hydrogel samples in tensile mode using an Instron 5567 universal testing machine. A 100 N load cell and a constant crosshead speed of 50 mm/min at 23 °C were used for drying tests. Rectangular specimens with length = 40 mm, width = 10 mm, thickness = 2–3 mm and a gauge length of 30 mm were used. Sandpaper was used to prevent slippage of the hydrogels in the grips of the tensile machine. The samples were stretched to different stretch ratios and held at constant strain while the water evaporated. During the evaporation, the engineering stress was monitored until all the water evaporated and the DN hydrogel became glassy.

### 4.5. Confined Compression Tests

Confined compression tests were performed with an Instron 5567 universal testing machine equipped with a 10 kN load cell, using a constant crosshead speed of 3 mm/min at room temperature. Disk-shaped samples with diameter = 35 mm and thickness = 4 mm were compressed in a custom-built transparent cylinder container with a plunger with clearance of 0.01 mm between the plunger and the container. 

## Figures and Tables

**Table 1:** DN hydrogels synthesized in this study.

DN Hydrogel	Formulation of AMPS and SAPS Gels (pphm) ^a^			Formulation of AAm Network (pphm) ^a^		
	OXGA	MBAA	UV Dose (J/cm^2^)	OXGA	MBAA	UV Dose (J/cm^2^)
AMPS(1,2,2,9)/ AAm(2,0.1,0.01,97)	2	2	9	0.1	0.01	97
AMPS(1,1,4,9)/ AAm(2,0.01,0.01,97)	1	4	9	0.01	0.01	97
SAPS(1,0.1,2,9)/ AAm(2,0.1,0,97)	0.1	2	9	0.1	0	97
SAPS(1,2,2,9)/ AAm(2,0.1,0,97)	2	2	9	0.1	0	97
SAPS(1,2,2,97)/ AAm(2,0.1,0.05,97)	2	2	97	0.1	0.05	97
SAPS(1,4,2,9,PEO)/ AAm(2,0.1,0.05,9)	4	2	9	0.1	0.05	9

^a^ parts per hundred parts monomer.

## Data Availability

The data that support the findings of this study are available from the corresponding author upon reasonable request.
